# Associations of urinary phytoestrogens with all-cause and cardiovascular mortality in adults: a population-based cohort study

**DOI:** 10.3389/fendo.2024.1400182

**Published:** 2024-09-10

**Authors:** Chao Xuan, Cong Zhao, Ting-Ting Zhou, Jun-Jie Guo, Deng Pan, Zi-Bo Wang, Guo-Wei He

**Affiliations:** ^1^ Department of Clinical Laboratory, The Affiliated Hospital of Qingdao University, Qingdao, China; ^2^ Department of Cardiology, The Affiliated Hospital of Qingdao University, Qingdao, China; ^3^ Department of Cardiovascular Surgery, TEDA International Cardiovascular Hospital, Academy of Medical Sciences & Peking Union Medical College, Tianjin, China; ^4^ Department of Surgery, Oregon Health and Science University, Portland, OR, United States

**Keywords:** urinary phytoestrogens, all-cause mortality, cardiovascular mortality, population-based cohort study, adult

## Abstract

**Background:**

The overall understanding of the correlations between mortality risk and phytoestrogens in general population remains limited. We examined the association between urinary phytoestrogen levels and all-cause and cardiovascular mortality based on the National Health and Nutrition Examination Survey (NHANES).

**Methods:**

Weighted Cox proportional hazard regression models were employed to calculate adjusted hazard ratios (HRs) and their 95% confidence intervals (CIs). Nonlinear relationships were assessed using multivariable-adjusted restricted cubic splines (RCS).

**Results:**

In the fully adjusted model, the highest quartiles of urinary genistein levels were correlated with significantly elevated all-cause (HR = 1.36, 95%CI: 1.16–1.59) and cardiovascular (HR = 1.58, 95%CI: 1.20–2.09) mortality. Urinary enterolactone levels in the third quartile were associated with reduced all-cause (HR = 0.77, 95%CI: 0.65–0.90) and cardiovascular (HR = 0.74, 95%CI: 0.55–0.99) mortality. In the highest quartiles of urinary daidzein levels, the cardiovascular mortality was significantly increased (HR = 1.44, 95%CI: 1.09–1.90). RCS showed an non-linear relationship between urinary daidzein levels and all-cause mortality (*P* = 0.04).

**Conclusion:**

In the context of a nationally representative sample, genistein exhibited associations with elevated all-cause and cardiovascular mortality, whereas enterolactone showed an association with reduced mortality. The dose–response relationship between urinary daidzein levels and all-cause mortality as well as sex-specific disparities in the impact of phytoestrogen levels should be considered.

## Introduction

Phytoestrogens, widely recognized as dietary estrogens, constitute a category of naturally occurring nonsteroidal compounds intrinsic to botanical species. These compounds are strikingly similar to 17β-estradiol, the primary hormone of the female endocrine system (1). Phytoestrogens are present in diverse components of daily diet, including fruits, vegetables, and beverages. Furthermore, these compounds have been detected in several botanical dietary supplements.

Phytoestrogens are classified into five distinct groups based on their chemical structures, namely, isoflavones, flavones, lignans, coumestans, and stilbenes (2). Of these, isoflavones and lignans are primary bioactive components and exert considerable physiological effects (3). Plant-derived isoflavones are present in the glucoside form. Their absorption in the human intestine is impeded by their high hydrophilicity and molecular weight. To enhance the estrogenic effects and bioavailability of isoflavones, they must undergo hydrolysis within the digestive tract by the intestinal mucosa with the aid of bacterial β-glucosidase, thereby leading to the formation of genistein and daidzein. Daidzein is further metabolized to produce two endogenous compounds, that is, O-desmethylangolensin (O-DMA) and equol (4). Lignans are plant-derived phenolic compounds that are biologically active and possess mild estrogenic properties. Lignans are abundantly available in flax seeds, sesame seeds, wheat germ, and rye (5). Once ingested, these compounds undergo conversion into enterolignan metabolites (enterodiol and enterolactone), catalyzed by bacterial enzymes within the mammalian digestive tract (6).

Phytoestrogens mimic the behavior of estrogens at low concentrations; however, at high concentrations, they inhibit estrogen receptor binding (7). This dual modulation signifies the complexity of their physiological effects. Their actions are primarily attributed to their interaction with two crucial estrogen receptors (ERs) (8) that vary in their tissue distribution. ERα is associated with cell proliferation and the mediation of physiological effects of estradiol, whereas ERβ counteracts the actions of ERα and is linked to antiproliferative and anti-inflammatory activities (9).

The plasma levels of phytoestrogens tend to vary among human populations. Asian populations, including the Chinese and Japanese, frequently consume soy products (10). People living in these areas have low incidence rates of menopausal symptoms, osteoporosis, cancer, and cardiovascular disease (CVD) (11). Therefore, these disparities have triggered investigations to comprehend the potential benefits of phytoestrogens along with studies on estrogen. Several studies have reported that phytoestrogens can induce beneficial biological effects in humans, such as anti-inflammatory, antioxidant, antineoplastic, and antiproliferative effects (12, 13). Nevertheless, some studies have identified inconsistencies in the observed beneficial impacts (14–16). These reports suggest that phytoestrogens have the capacity to emulate estradiol, binding to steroid receptors within reproductive tissues, thereby instigating endocrine perturbations and potentially facilitating tumor cell proliferation and metastasis. This variability in the functions of phytoestrogens is likely to be dose dependent (17). Estimating phytoestrogen levels in the urine appears to be an acceptable method for objectively and precisely evaluating phytoestrogen exposure. The National Health and Nutrition Examination Survey (NHANES) assessed urinary phytoestrogen levels in sampled populations across the United States in six consecutive cycles from 1999 to 2010, covering a 12-year period. This survey provided us with the opportunity to explore the correlation between urinary phytoestrogen levels and all-cause and cardiovascular mortality.

## Participants and methods

### Study population

NHANES is a program of the United States that includes a range of carefully crafted comprehensive studies, all geared toward assessing the nutritional status and physical health. This effort denotes a nationally representative, large-scale, multistage health survey that continues to be conducted among the civilian noninstitutionalized population. NHANES is a prominent program under the auspices of the National Center for Health Statistics (NCHS), a key division within the Centers for Disease Control and Prevention (CDC). NCHS is responsible for generating the necessary health-related statistics for the U.S. The protocols of NHANES are approved by the Ethics Review Board of NCHS to protect the well-being and rights of all participants. All participants provided written informed consent before undergoing the interview and examination (18).

This is a population-based cohort study. Data extracted from six NHANES survey cycles spanning 1999–2010, with each 2-year period constituting a cycle, were integrated with the mortality data sourced from the National Death Index (NDI) until December 31, 2019, to establish the foundation for this cohort study. The NHANES survey conducted from 1999 to 2010 included a total of 62,160 respondents. After excluding individuals for whom urinary phytoestrogen data (n = 22,634) were not available, were under 20 years of age (n = 29,696), and lacked follow-up information (n = 47), the analysis comprised a final cohort of 9,783 participants

### Phytoestrogen measurement

Urine samples were handled, preserved, and transported to the Division of Laboratory Sciences by the National Center for Environmental Health (NCEH). The center operates under the auspices of the CDC, and its primary mission is to perform comprehensive analyses. For detailed information on sample collection and processing, please refer to the Laboratory/Medical Technologists section of the NHANES Manual (19). Until analysis in the laboratory of NCEH, the sample vials were stored at -20°C. Laboratory data from NHANES comprised measurements of urinary levels for six phytoestrogens, namely, equol, genistein, O-DMA, daidzein, enterolactone, and enterodiol. Reversed-phase high-performance liquid chromatography with atmospheric pressure photoionization-tandem mass spectrometry (HPLC-APCI-MS/MS) was used to perform quantitative analysis in three survey cycles from 1999 to 2004. High-performance liquid chromatography with atmospheric pressure photoionization-tandem mass spectrometry (HPLC-APPI-MS/MS) was utilized in three subsequent cycles from 2005 to 2010 (20, 21). Crossover studies comparing samples analyzed by the two methods showed high correlation coefficients (r>0.99) and regression slopes close to 1 and intercepts close to 0. Urinary creatinine levels were estimated using the Jaffé rate reaction, and the levels of urinary phytoestrogens were corrected with urinary creatinine.

### Ascertaining the outcome

The NCHS integrated data were collected from multiple NCHS population surveys, and mortality records were obtained from the NDI. The vital statuses of participants were determined up to December 31, 2019 (22). This study focused on cardiovascular and all-cause mortality. Cardiovascular mortality referred to fatalities attributed to heart diseases and stroke as per the guidelines of the International Statistical Classification of Diseases (23).

### Covariates

Demographic data were gathered to obtain details about the following sociodemographic characteristics: gender, age, race/ethnicity, family poverty income ratio (PIR), marital status, and educational levels. The participants’ smoking status was categorized based on serum cotinine levels (less than the limit of detection (LOD), LOD–10 ng/mL, and >10 ng/mL). Data pertaining to waist circumference, heart rates, and body mass index (BMI) were obtained from the examination results. The levels of C-reactive protein (CRP), high density lipoprotein cholesterol (HDL-C), total cholesterol (TCHO), alanine aminotransferase (ALT), glucose (GLU), triglycerides (TG), serum creatinine (SCr), Albumin (ALB) and urinary phytoestrogens were fetched from laboratory findings. Hypertension was defined as self-reporting a doctor-diagnosed high blood pressure, taking prescribed hypertension medication, and blood pressure of ≥140/90 mmHg (averaged over multiple assessments). Diabetes mellitus (DM) was delineated by the following criteria: clinical diagnosis by healthcare professionals, present use of insulin, fasting blood glucose concentration equal to or exceeding 7.0 mmol/L, a 2-hour postprandial glucose concentration equal to or exceeding 11.1 mmol/L, and/or a glycated hemoglobin level equal to or exceeding 6.5%

### Statistical analysis

As per NCHS guidelines, sample weights and taylor series linearization methods to estimate the variance in all NHANES surveys conducted between 1999 and 2010, which ensured that precise national estimates were generated. Masked variance units were obtained from demographic data files (24).

The R (version 4.3.1) and the Stata Statistical Software (Version 16.0) were used to perform all statistical analyses. Continuous variables were presented as mean ± standard errors (SE) and categorical variables as proportions and 95% confidence intervals (CIs). Categorical variables were analyzed using chi-square test or Fisher’s exact probability test. The independent sample *t*-test was employed to compare two sample means from unrelated groups. Furthermore, one-way analysis of variance was used to compare the means among three or more unrelated groups. For *post-hoc* testing with multiple comparisons, the least significant difference method was used. The correlations between urinary phytoestrogen levels and covariates were examined using Spearman correlation analysis, the results of which were depicted visually in the form of a heatmap. The corrected urinary phytoestrogen levels were classified into four quartiles (Q1–Q4). In addition, hazard ratios (HRs) and their corresponding 95% CIs were estimated to evaluate all-cause and cardiovascular mortality across various quartiles of urinary phytoestrogen levels. Weighted Cox proportional hazard regression models were used for this purpose. Four statistical models were fitted. Model 1 was adjusted for age, sex, race/ethnicity, educational level, family PIR, and marital status. Model 2, on the contrary, was further adjusted for BMI, waist circumference, and heart rates, whereas Model 3 was further adjusted for serum cotinine levels, drinking status, and presence of hypertension and DM. Finally, Model 4 was further adjusted for history of cardiovascular events, serum levels of CRP, TCHO, ALT, GLU, TG, ALB, SCr, and HDL-C. To clarify the dose–response relationship between corrected urinary phytoestrogen levels and mortality, restricted cubic spline regression model with four knots was applied. Nonlinearity was evaluated using the likelihood ratio test. Multiple imputation was used to mitigate the reduction in sample size due to missing covariates. Stratified analyses based on sex (men or women) and age (>55 years in women) were performed. A sensitivity analysis was performed by excluding mortality data from the initial 2 years of the follow-up period. Two-sided *P* < 0.05 was considered statistically significant.

## Results

### Baseline characteristics of study participants

**Figure 1:**
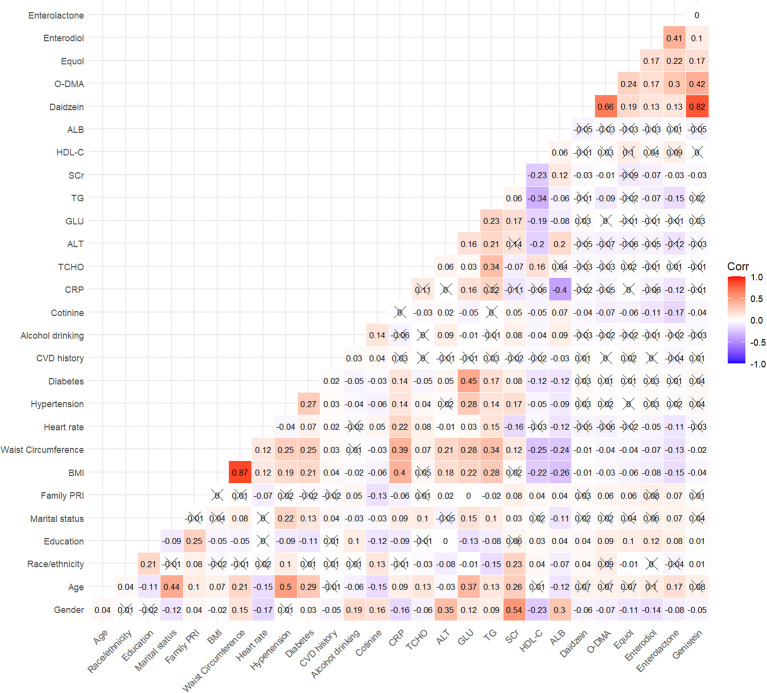
Spearman correlation matrix of urinary phytoestrogens in the study. The values of correlation coefficient contained in a matrix are represented as colors and an”×”indicates that the correlation was not significant (*P* > 0.05).

In this study, 9,783 adult participants were included, with a weighted mean (SE) age of 46.34 (0.27) years, of whom 4,692 were men (weighted proportion: 48.03%). **Table 1** presented the baseline demographic and clinical parameters of participants in the survival, all-cause mortality, and cardiovascular mortality groups. Among the urinary phytoestrogens, the all-cause mortality population exhibited significantly reduced levels of urinary daidzein (weighted: 250.53 ± 23.12 µg/g vs. 354.90 ± 23.62 µg/g, *P* < 0.001), O-DMA (weighted: 53.81 ± 6.66 µg/g vs. 86.21 ± 7.09 µg/g, *P* = 0.022), equol (weighted: 42.44 ± 11.90 µg/g vs. 60.67 ± 6.21 µg/g, *P* = 0.023), and genistein (weighted: 127.64 ± 13.13 µg/g vs. 166.27 ± 12.24 µg/g, *P* = 0.018). These reductions were not observed in a population with cardiovascular mortality. In addition, the baseline demographic and clinical parameters of the participants, which were categorized into quartiles based on the urinary phytoestrogen levels, are summarized in **Supplementary Table S1**. The heat map of Spearman correlation coefficients between serum phytoestrogens levels and covariates is shown in **Figure 1**.

**Table 1:** Baseline demographic and clinical parameters among participants in survival, all-cause mortality, and cardiovascular mortality group.

	All (n = 9783)	Survival (n = 7706)	All-Cause Mortality (n = 2077)	Cardiovascular Mortality (n = 668)	*P*_1_^*^	*P*_2_^**^
Age (years)	46.34 ± 0.27	42.60 ± 0.25	66.43 ± 0.47	69.30 ± 0.76	<0.001	<0.001
Gender, Male (%)	48.03(46.80-49.26)	47.42(46.11-48.73)	51.32(48.52-54.11)	52.67(48.37-56.93)	0.010	0.021
Race/ethnicity (%)						
Mexican American	7.95(6.76-9.33)	8.69(7.43-10.13)	3.99(2.99-5.31)	4.05(2.67-6.11)	<0.001	<0.001
Other Hispanic	5.11(3.82-6.81)	5.34(4.07-6.98)	3.87(2.08-7.12)	2.03(0.97-4.21)		
Non-Hispanic White	70.53(67.86-73.06)	68.97(66.23-71.59)	78.87(75.54-81.85)	79.96(75.29-83.93)		
Non-Hispanic Black	11.16(9.78-12.70)	11.22(9.82-12.8)	10.79(9.19-12.64)	11.00(8.62-13.93)		
Other Race	5.26(4.51-6.13)	5.78(4.94-6.76)	2.47(1.65-3.69)	2.96(1.44-5.96)		
Education (%)						
< High school	19.26(17.94-20.65)	16.85(15.64-18.13)	32.20(28.81-35.78)	38.17(32.60-44.07)	<0.001	<0.001
High school/GED	25.18(23.74-26.68)	24.96(23.42-26.56)	26.41(24.11-28.86)	25.34(21.21-29.97)		
> High school	55.56(53.56-57.54)	58.2(56.19-60.18)	41.39(37.82-45.05)	36.49(31.85-41.40)		
Marital Status (%)						
Divorced/widowed/separated	16.24(14.85-17.73)	18.04(16.47-19.72)	6.61(5.42-8.03)	6.07(4.33-8.47)	<0.001	<0.001
Married/unmarried couple	65.26(63.64-66.84)	66.75(64.97-68.49)	57.22(54.48-59.92)	55.94(50.34-61.40)		
Never married	18.50(17.32-19.74)	15.21(14.05-16.45)	36.17(33.58-38.84)	37.98(32.84-43.40)		
Family PRI						
<1	12.58(11.38-13.87)	12.23(11.06-13.51)	14.42(12.33-16.81)	13.85(11.43-16.69)	0.021	0.268
>1	87.42(86.13-88.62)	87.77(86.49-88.94)	85.58(83.19-87.67)	86.15(83.31-88.57)		
BMI (kg/m^2^)	28.42 ± 0.10	28.41 ± 0.11	28.45 ± 0.18	28.64 ± 0.34	<0.001	0.085
Waist Circumference (cm)	97.28 ± 0.25	96.60 ± 0.27	100.94 ± 0.41	102.07 ± 0.78	<0.001	<0.001
Heart rate (bpm)	72.83 ± 0.15	72.82 ± 0.17	72.88 ± 0.36	71.39 ± 0.62	0.125	<0.001
Hypertension (%)	35.04(33.76-36.33)	29.17(27.91-30.47)	66.53(63.83-69.12)	74.05(69.40-78.22)	<0.001	<0.001
Diabetes (%)	10.78(10.02-11.58)	7.92(7.29-8.59)	26.14(23.96-28.44)	31.68(27.51-36.16)	<0.001	<0.001
Alcohol drinking (%)	89.71(88.45-90.86)	90.45(89.10-91.66)	85.74(83.34-87.84)	85.25(81.73-88.19)	<0.001	0.001
CVD history (%)	14.02(12.95-15.15)	13.92(12.84-15.08)	14.55(12.50-16.87)	24.95(21.09-29.25)	0.576	<0.001
Cotinine (ng/mL)						
< LOD	16.07(14.57-17.69)	16.02(14.50-17.65)	16.35(14.14-18.83)	15.20(12.20-18.77)	0.890	0.500
LOD-10	56.86(55.26-58.45)	56.96(55.27-58.64)	56.30(53.47-59.08)	60.17(54.81-65.29)		
> 10	27.07(25.52-28.68)	27.02(25.36-28.74)	27.35(24.90-29.96)	24.64(20.06-29.87)		
CRP (mg/dL)	0.426 ± 0.008	0.392 ± 0.009	0.611 ± 0.029	0.545 ± 0.036	<0.001	<0.001
TCHO (mmol/L)	5.18 ± 0.02	5.16 ± 0.02	5.26 ± 0.04	5.21 ± 0.07	0.296	0.660
ALT (U/L)	26.09 ± 0.26	26.44 ± 0.26	24.23 ± 0.69	22.57 ± 0.94	0.007	<0.001
GLU (mmol/L)	5.36 ± 0.02	5.25 ± 0.02	5.97 ± 0.07	6.17 ± 0.11	<0.001	<0.001
TG (mmol/L)	1.72 ± 0.02	1.70 ± 0.03	1.83 ± 0.05	1.85 ± 0.06	0.107	0.441
SCr (umol/L)	76.57 ± 0.31	74.97 ± 0.30	85.19 ± 0.92	90.15 ± 2.02	<0.001	<0.001
HDL-C (mmol/L)	1.77 ± 0.02	1.74 ± 0.02	1.91 ± 0.03	1.91 ± 0.05	<0.001	<0.001
ALB (g/L)	42.90 ± 0.06	43.07 ± 0.07	41.93 ± 0.09	41.98 ± 0.13	<0.001	<0.001
Daidzein (µg/g creatinine)	338.51 ± 19.89	354.90 ± 23.62	250.53 ± 23.12	332.94 ± 68.91	<0.001	0.159
O-DMA (µg/g creatinine)	81.12 ± 6.05	86.21 ± 7.09	53.81 ± 6.66	73.27 ± 17.02	0.022	0.667
Equol (µg/g creatinine)	57.81 ± 6.09	60.67 ± 6.21	42.44 ± 11.90	44.00 ± 10.42	0.023	0.771
Enterodiol (µg/g creatinine)	134.63 ± 9.32	136.42 ± 10.32	125.00 ± 14.40	123.67 ± 29.15	0.353	0.557
Enterolactone (µg/g creatinine)	790.63 ± 30.21	796.24 ± 34.34	760.51 ± 35.44	826.03 ± 63.87	0.730	0.381
Genistein (µg/g creatinine)	160.20 ± 10.41	166.27 ± 12.24	127.64 ± 13.13	167.13 ± 41.00	0.018	0.684

GED, General Educational Development; PIR, Poverty Income Ratio; BMI, Body Mass Index; CVD, Cardiovascular Disease LOD: The limit of detection; CRP, C-reactive Protein; TCHO, Total Cholesterol; ALT, Alanine Aminotransferase; GLU, Glucose; TG, Triglycerides; SCr, Serum Creatinine; HDL-C, High Density Lipoprotein Cholestero; ALB, Albumin; O-DMA, O-desmethylangolensin.

*P values for comparisons of variables between Survival and All-Cause Mortality groups.

**P values for comparisons of variables between Survival and Cardiovascular Mortality groups.

### Urinary phytoestrogen levels and all-cause mortality

**Figure 2:**
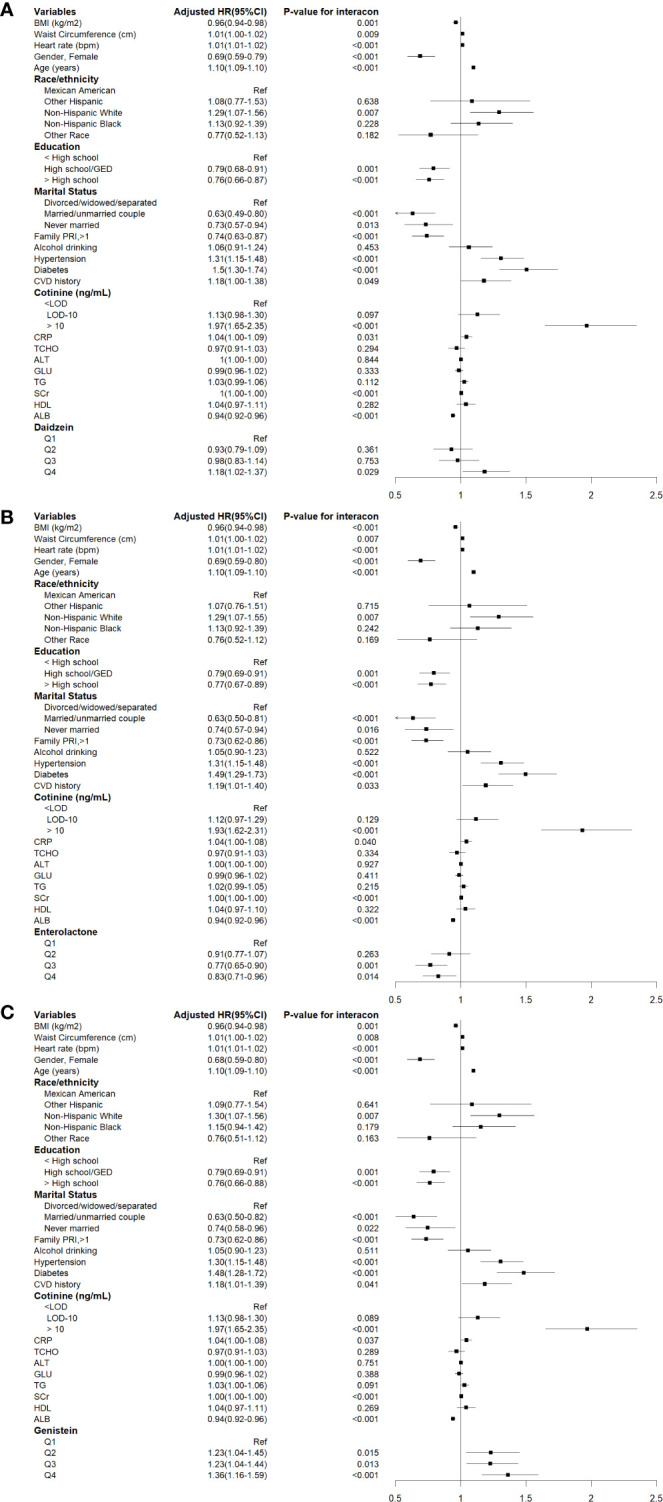
Cox proportional hazards analysis for the associations between urinary phytoestrogens levels and all-cause mortality from NHANES 1999-2010. Data are expressed as hazard ratio (HR) and 95% Confidence Interval (CI). **(A)** Daidzein, **(B)** Enterolactone, **(C)** Genistein. The results were adjusted for gender, age, race/ethnicity, educational attainment, marital status, family PIR, BMI, waist circumference, heart rates, serum cotinine levels, drinking status, presence of diabetes and hypertension, history of cardiovascular events, serum levels of CRP, TCHO, ALT, GLU, TG, ALB, SCr, and HDL-C.

During a median follow-up of 14.45 years (95% CI: 14.30-14.60), a total of 2077 cases of all-cause mortality were recorded, of which 668 cases were attributable to cardiovascular mortality. The associations between urinary phytoestrogen levels and all-cause mortality using weighted Cox proportional regression models adjusted for covariates are shown in **Table 2**, and **Figure 2** illustrates the corresponding forest plots. The low concentration groups served as reference categories. Elevated urinary daidzein levels were associated with increased all-cause mortality, with a 18% increased risk among participants in the highest quartile (HR = 1.18, 95% CI: 1.02-1.37, *P* = 0.029). Similarly, compared with the lowest quartile of urinary genistein levels, the second (HR = 1.23, 95%CI: 1.04-1.45, *P* = 0.015), third (HR = 1.23, 95%CI: 1.04-1.44, *P* = 0.013), and fourth (HR = 1.36, 95%CI: 1.16-1.59, *P* < 0.001) quartiles showed an increased risk of all-cause mortality. In contrast, the third (HR = 0.77, 95% CI: 0.65-0.90, *P* = 0.001) and fourth (HR = 0.83, 95% CI: 0.71-0.96, *P* = 0.014) quartiles of urinary enterolactone levels were associated with significantly decreased all-cause mortality. All-cause mortality was not associated with three other phytoestrogens: O-DMA, equol, and enterodiol.

**Table 2:** Hazard ratios for all-cause mortality of all participants, stratified by corrected urinary phytoestrogens levels quartiles.

	All-cause Mortality (HR and 95%CI)									
	Crude	*P*	Model 1	*P*	Model 2	*P*	Model 3	*P*	Model 4	*P*
Daidzein (µg/g creatinine)										
Q1 (<16.87)	1(Reference)		1(Reference)		1(Reference)		1(Reference)		1(Reference)	
Q2 (16.87-49.19)	1.00(0.83-1.19)	0.971	0.90(0.76-1.07)	0.238	0.92(0.78-1.09)	0.325	0.91(0.77-1.07)	0.253	0.93(0.79-1.09)	0.361
Q3 (49.19-170.08)	**1.16(1.00-1.36)**	**0.049**	0.96(0.82-1.13)	0.643	0.98(0.84-1.14)	0.771	0.95(0.81-1.11)	0.494	0.98(0.83-1.14)	0.753
Q4 (≥170.08)	**1.26(1.07-1.47)**	**0.005**	1.14(0.99-1.31)	0.066	**1.17(1.03-1.34)**	**0.019**	**1.15(1.01-1.31)**	**0.041**	**1.18(1.02-1.37)**	**0.029**
O-DMA (µg/g creatinine)										
Q1 (<0.61)	1(Reference)		1(Reference)		1(Reference)		1(Reference)		1(Reference)	
Q2 (0.61-3.00)	**1.22(1.03-1.45)**	**0.025**	1.03(0.87-1.23)	0.727	1.03(0.86-1.23)	0.731	1.01(0.84-1.21)	0.918	1.02(0.87-1.20)	0.782
Q3 (3.00-18.93)	1.15(1.00-1.33)	0.052	0.96(0.82-1.12)	0.574	0.96(0.82-1.13)	0.643	0.97(0.83-1.14)	0.741	0.98(0.84-1.15)	0.825
Q4 (≥18.93)	1.15(0.98-1.34)	0.077	1.04(0.89-1.22)	0.604	1.08(0.93-1.27)	0.303	1.09(0.93-1.28)	0.262	1.10(0.95-1.29)	0.202
Equol (µg/g creatinine)										
Q1 (<3.02)	1(Reference)		1(Reference)		1(Reference)		1(Reference)		1(Reference)	
Q2 (3.02-6.61)	1.11(0.90-1.35)	0.328	0.91(0.76-1.0)	0.321	0.93(0.78-1.12)	0.455	0.93(0.78-1.1)	0.405	0.95(0.81-1.11)	0.519
Q3 (6.61-14.15)	1.17(0.99-1.38)	0.072	0.87(0.72-1.06)	0.163	0.88(0.73-1.07)	0.198	0.88(0.73-1.05)	0.156	0.89(0.76-1.04)	0.139
Q4 (≥14.15)	**1.20(1.01-1.42)**	**0.038**	0.89(0.74-1.07)	0.222	0.92(0.76-1.10)	0.360	0.93(0.77-1.11)	0.404	0.97(0.82-1.14)	0.682
Enterodiol (µg/g creatinine)										
Q1 (<14.48)	1(Reference)		1(Reference)		1(Reference)		1(Reference)		1(Reference)	
Q2 (14.48-38.00)	1.06(0.88-1.28)	0.536	0.96(0.81-1.14)	0.673	0.96(0.81-1.13)	0.618	0.96(0.81-1.14)	0.638	0.94(0.81-1.10)	0.443
Q3 (38.00-91.76)	1.08(0.93-1.27)	0.306	0.88(0.74-1.03)	0.106	0.88(0.75-1.04)	0.126	0.89(0.76-1.05)	0.154	0.88(0.75-1.03)	0.106
Q4 (≥91.76)	1.15(0.96-1.38)	0.120	0.98(0.82-1.17)	0.802	1.00(0.84-1.19)	0.989	1.01(0.84-1.2)	0.945	1.00(0.85-1.17)	0.997
Enterolactone (µg/g creatinine)										
Q1 (<100.24)	1(Reference)		1(Reference)		1(Reference)		1(Reference)		1(Reference)	
Q2 (100.24-343.28)	1.09(0.91-1.29)	0.345	0.88(0.75-1.03)	0.108	0.9(0.77-1.06)	0.208	0.95(0.81-1.10)	0.485	0.91(0.77-1.07)	0.263
Q3 (343.28-825.81)	1.16(0.98-1.36)	0.076	**0.74(0.64-0.86)**	**<0.001**	**0.74(0.64-0.86)**	**<0.001**	**0.77(0.67-0.89)**	**<0.001**	**0.77(0.65-0.90)**	**0.001**
Q4 (≥825.81)	**1.34(1.14-1.59)**	**0.001**	**0.75(0.64-0.87)**	**<0.001**	**0.78(0.67-0.92)**	**0.003**	**0.82(0.70-0.96)**	**0.014**	**0.83(0.71-0.96)**	**0.014**
Genistein (µg/g creatinine)										
Q1 (<8.69)	1(Reference)		1(Reference)		1(Reference)		1(Reference)		1(Reference)	
Q2 (8.69-23.13)	**1.43(1.22-1.68)**	**<0.001**	1.27(1.08-1.50)	0.005	**1.25(1.06-1.48)**	**0.009**	**1.22(1.04-1.43)**	**0.015**	**1.23(1.04-1.45)**	**0.015**
Q3 (23.13-79.19)	**1.54(1.33-1.79)**	**<0.001**	1.27(1.09-1.48)	0.002	**1.27(1.1-1.47)**	**0.001**	**1.22(1.05-1.41)**	**0.010**	**1.23(1.04-1.44)**	**0.013**
Q4 (≥79.19)	**1.57(1.35-1.82)**	**<0.001**	1.36(1.17-1.58)	<0.001	**1.38(1.19-1.6)**	**<0.001**	**1.34(1.15-1.55)**	**<0.001**	**1.36(1.16-1.59)**	**<0.001**

HR, Hazard Ratios; 95% CI, 95% Confidence Intervals; O-DMA, O-desmethylangolensin.

Bold values denote statistical significance at the P < 0.05 level.

### Urinary phytoestrogen levels and cardiovascular mortality

**Figure 3:**
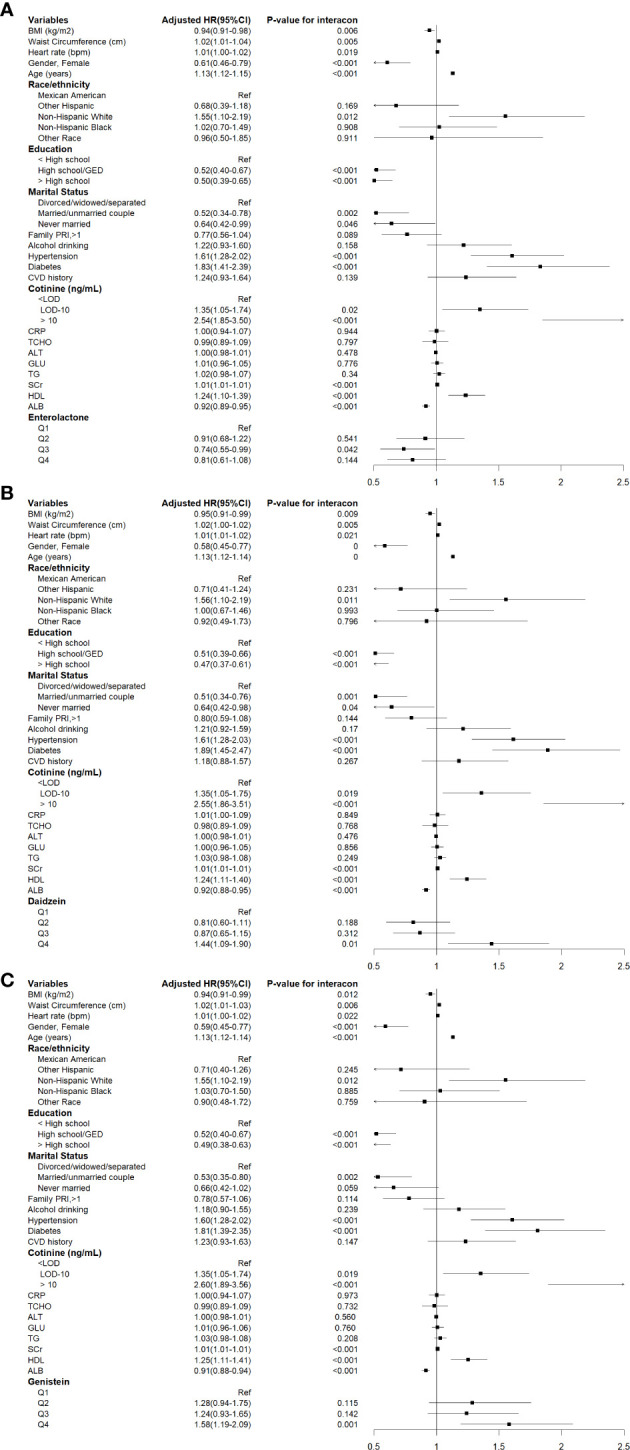
Cox proportional hazards analysis for the association between urinary phytoestrogens levels and cardiovascular mortality from NHANES 1999-2010. Data are expressed as hazard ratio (HR) and 95% Confidence Interval (CI). **(A)** Daidzein, **(B)** Enterolactone, **(C)** Genistein. The results were adjusted for gender, age, race/ethnicity, educational attainment, marital status, family PIR, BMI, waist circumference, heart rates, serum cotinine levels, drinking status, presence of diabetes and hypertension, history of cardiovascular events, serum levels of CRP, TCHO, ALT, GLU, TG, ALB, SCr, and HDL-C.

The associations between corrected urinary phytoestrogen levels and cardiovascular mortality are listed in **Table 3**, and the corresponding forest plots are illustrated in **Figure 3**. The cardiovascular mortality was significantly increased in the highest quartiles of urinary daidzein (HR = 1.44, 95% CI: 1.09–1.90, *P* = 0.010) and of genistein (HR = 1.58, 95% CI: 1.20–2.09, *P* = 0.001) levels. Urinary enterolactone showed an association with reduced cardiovascular mortality risk when the levels were within the third quartile (HR = 0.74, 95% CI: 0.55–0.99, *P* = 0.042).

**Table 3:** Hazard ratios for cardiovascular mortality of all participants, stratified by corrected urinary phytoestrogens levels quartiles.

	Cardiovascular Mortality (HR and 95%CI)									
	Crude	*P*	Model 1	*P*	Model 2	*P*	Model 3	*P*	Model 4	*P*
Daidzein (µg/g creatinine)										
Q1 (<16.87)	1(Reference)		1(Reference)		1(Reference)		1(Reference)		1(Reference)	
Q2 (16.87-49.19)	0.97(0.69-1.37)	0.876	0.75(0.54-1.04)	0.086	0.75(0.54-1.05)	0.090	0.76(0.54-1.05)	0.098	0.81(0.60-1.11)	0.188
Q3 (49.19-170.08)	1.19(0.90-1.59)	0.225	0.88(0.66-1.18)	0.384	0.89(0.67-1.18)	0.413	0.83(0.62-1.12)	0.231	0.87(0.65-1.15)	0.312
Q4 (≥170.08)	**1.69(1.26-2.28)**	**0.001**	**1.33(1.01-1.74)**	**0.041**	**1.35(1.02-1.78)**	**0.033**	**1.32(1.02-1.72)**	**0.036**	**1.44(1.09-1.90)**	**0.010**
O-DMA (µg/g creatinine)										
Q1 (<0.61)	1(Reference)		1(Reference)		1(Reference)		1(Reference)		1(Reference)	
Q2 (0.61-3.00)	0.93(0.67-1.28)	0.649	0.76(0.56-1.03)	0.078	0.77(0.57-1.04)	0.086	0.74(0.55-1.01)	0.060	0.75(0.56-1.02)	0.066
Q3 (3.00-18.93)	1.15(0.87-1.51)	0.321	0.87(0.65-1.16)	0.328	0.85(0.64-1.15)	0.291	0.86(0.64-1.14)	0.293	0.87(0.66-1.15)	0.323
Q4 (≥18.93)	1.28(0.96-1.70)	0.094	1.11(0.86-1.43)	0.430	1.15(0.89-1.47)	0.278	1.11(0.87-1.42)	0.385	1.10(0.83-1.46)	0.501
Equol (µg/g creatinine)										
Q1 (<3.02)	1(Reference)		1(Reference)		1(Reference)		1(Reference)		1(Reference)	
Q2 (3.02-6.61)	1.09(0.77-1.54)	0.628	0.88(0.62-1.26)	0.480	0.89(0.62-1.28)	0.527	0.93(0.66-1.30)	0.652	0.93(0.68-1.27)	0.642
Q3 (6.61-14.15)	1.21(0.93-1.57)	0.145	0.80(0.59-1.08)	0.141	0.79(0.58-1.07)	0.127	0.81(0.61-1.07)	0.138	0.83(0.62-1.10)	0.187
Q4 (≥14.15)	**1.35(1.03-1.77)**	**0.030**	0.90(0.67-1.2)	0.455	0.91(0.68-1.21)	0.498	0.92(0.70-1.22)	0.556	0.96(0.72-1.28)	0.795
Enterodiol (µg/g creatinine)										
Q1 (<14.48)	1(Reference)		1(Reference)		1(Reference)		1(Reference)		1(Reference)	
Q2 (14.48-38.00)	1.23(0.92-1.65)	0.159	1.06(0.81-1.40)	0.673	1.04(0.79-1.36)	0.785	1.05(0.79-1.38)	0.752	1.00(0.75-1.32)	0.989
Q3 (38.00-91.76)	1.17(0.90-1.52)	0.227	0.84(0.64-1.11)	0.223	0.83(0.63-1.09)	0.180	0.85(0.64-1.13)	0.258	0.86(0.65-1.13)	0.273
Q4 (≥91.76)	1.25(0.93-1.67)	0.140	0.91(0.66-1.25)	0.537	0.91(0.66-1.26)	0.576	0.92(0.66-1.27)	0.610	0.92(0.69-1.24)	0.593
Enterolactone (µg/g creatinine)										
Q1 (<100.24)	1(Reference)		1(Reference)		1(Reference)		1(Reference)		1(Reference)	
Q2 (100.24-343.28)	1.12(0.82-1.53)	0.464	0.91(0.7-1.18)	0.457	0.94(0.72-1.22)	0.622	0.99(0.77-1.28)	0.942	0.91(0.68-1.22)	0.541
Q3 (343.28-825.81)	1.25(0.97-1.59)	0.078	**0.70(0.55-0.89)**	**0.004**	**0.70(0.56-0.89)**	**0.004**	**0.76(0.60-0.96)**	**0.021**	**0.74(0.55-0.99)**	**0.042**
Q4 (≥825.81)	**1.51(1.17-1.95)**	**0.002**	**0.75(0.58-0.97)**	**0.027**	0.79(0.61-1.02)	0.070	0.84(0.66-1.07)	0.162	0.81(0.61-1.08)	0.108
Genistein (µg/g creatinine)										
Q1 (<8.69)	1(Reference)		1(Reference)		1(Reference)		1(Reference)		1(Reference)	
Q2 (8.69-23.13)	**1.52(1.16-1.98)**	**0.003**	**1.32(1.00-1.75)**	**0.049**	1.32(1.00-1.74)	0.053	1.20(0.90-1.61)	0.216	1.28(0.94-1.75)	0.115
Q3 (23.13-79.19)	**1.61(1.17-2.21)**	**0.004**	**1.35(1.02-1.77)**	**0.034**	**1.35(1.03-1.77)**	**0.029**	1.20(0.90-1.60)	0.201	1.24(0.93-1.65)	0.142
Q4 (≥79.19)	**1.97(1.50-2.60)**	**<0.001**	**1.58(1.23-2.03)**	**<0.001**	**1.61(1.26-2.06)**	**<0.001**	**1.46(1.14-1.88)**	**0.003**	**1.58(1.20-2.09)**	**0.001**

HR, Hazard Ratios; 95% CI, 95% Confidence Intervals; O-DMA, O-desmethylangolensin.

Bold values denote statistical significance at the P < 0.05 level.

### Stratification analyses

The sex-specific relationship between urinary phytoestrogen excretion and all-cause and cardiovascular mortality are shown in **Table 4**. The third quartile of urinary enterolactone levels showed an association with reduced risk of all-cause mortality in women (HR = 0.72, 95% CI: 0.56–0.92, *P* = 0.008). On the contrary, when assessing the highest quartile of urinary genistein levels against the first quartile, the all-cause mortality was significantly elevated in both men (HR = 1.30, 95% CI: 1.05–1.61, *P* = 0.015) and women (HR = 1.42, 95% CI: 1.13–1.80, *P* = 0.003). There was no discernible link between any of the phytoestrogens and cardiovascular mortality in men. Nonetheless, in women, significantly elevated urinary daidzein (HR = 1.73, 95% CI: 1.13–2.65, *P* = 0.012) and genistein (HR = 2.06, 95% CI: 1.34–3.20, *P* = 0.001) levels in the highest quartile were linked to an increased cardiovascular mortality compared with the lowest quartile. An association with reduced cardiovascular mortality risk was observed when the urinary enterolactone levels were within the third quartile (HR: 0.60, 95% CI: 0.39–0.93, *P* = 0.021) in women.

**Table 4:** The sex-specific relationships between urinary phytoestrogens levels and all-cause and cardiovascular mortality.

	All-cause Mortality (HR and 95%CI)				Cardiovascular Mortality (HR and 95%CI)			
	Male		Female		Male		Female	
	Multivariable Model	*P*	Multivariable Model	*P*	Multivariable Model	*P*	Multivariable Model	*P*
Daidzein (µg/g cratinine)								
Q1 (<16.87)	1 [Reference]		1 [Reference]		1 [Reference]		1 [Reference]	
Q2 (16.87-49.19)	1.02(0.82-1.26)	0.974	0.86(0.67-1.09)	0.202	0.88(0.59-1.31)	0.528	0.80(0.9-1.32)	0.390
Q3 (49.19-170.08)	0.90(0.73-1.10)	0.305	1.08(0.85-1.37)	0.542	0.77(0.53-1.13)	0.181	1.05(0.68-1.61)	0.831
Q4 (≥170.08)	1.15(0.95-1.42)	0.154	1.20(0.95-1.51)	0.120	1.27(0.86-1.87)	0.237	**1.73(1.13-2.65)**	**0.012**
O-DMA (µg/g cratinine)								
Q1 (<0.61)	1 [Reference]		1 [Reference]		1 [Reference]		1 [Reference]	
Q2 (0.61-3.00)	0.94(0.76-1.16)	0.552	1.15(0.91-1.46)	0.250	0.81(0.54-1.22)	0.311	0.71(0.44-1.14)	0.161
Q3 (3.00-18.93)	1.03(0.84-1.27)	0.759	0.96(0.75-1.24)	0.778	1.04(0.72-1.49)	0.850	0.77(0.50-1.20)	0.250
Q4 (≥18.93)	1.05(0.85-1.31)	0.655	1.17(0.93-1.48)	0.178	1.11(0.75-1.64)	0.590	1.10(0.72-1.69)	0.660
Equol (µg/g cratinine)								
Q1 (<3.02)	1 [Reference]		1 [Reference]		1 [Reference]		1 [Reference]	
Q2 (3.02-6.61)	0.98(0.78-1.22)	0.836	0.95(0.74-1.20)	0.654	1.12(0.74-1.71)	0.592	0.82(0.50-1.34)	0.422
Q3 (6.61-14.15)	1.03(0.83-1.27)	0.809	0.80(0.63-1.03)	0.083	0.99(0.67-1.47)	0.978	0.76(0.49-1.17)	0.206
Q4 (≥14.15)	0.97(0.78-1.21)	0.810	0.97(0.76-1.24)	0.805	1.13(0.76-1.68)	0.539	0.90(0.58-1.40)	0.635
Enterodiol (µg/g cratinine)								
Q1 (<14.48)	1 [Reference]		1 [Reference]		1 [Reference]		1 [Reference]	
Q2 (14.48-38.00)	0.93(0.76-1.14)	0.470	0.99(0.78-1.25)	0.930	0.98(0.68-1.42)	0.929	1.07(0.67-1.69)	0.778
Q3 (38.00-91.76)	0.85(0.69-1.05)	0.124	0.94(0.75-1.19)	0.610	0.84(0.58-1.21)	0.353	0.95(0.62-1.46)	0.824
Q4 (≥91.76)	1.06(0.86-1.32)	0.585	1.00(0.79-1.26)	0.970	1.13(0.75-1.71)	0.545	0.92(0.60-1.41)	0.692
Enterolactone (µg/g cratinine)								
Q1 (<100.24)	1 [Reference]		1 [Reference]	1.000	1 [Reference]		1 [Reference]	
Q2 (100.24-343.28)	0.90(0.73-1.12)	0.367	0.97(0.76-1.24)	0.807	1.10(0.72-1.67)	0.666	0.83(0.54-1.27)	0.383
Q3 (343.28-825.81)	0.82(0.67-1.00)	0.060	**0.72(0.56-0.92)**	**0.008**	0.92(0.61-1.38)	0.683	**0.60(0.39-0.93)**	**0.021**
Q4 (≥825.81)	0.82(0.66-1.01)	0.066	0.87(0.70-1.09)	0.216	0.99(0.66-1.49)	0.974	0.71(0.48-1.06)	0.095
Genistein (µg/g creatinine)								
Q1 (<8.69)	1 [Reference]		1 [Reference]		1 [Reference]		1 [Reference]	
Q2 (8.69-23.13)	1.25(1.00-1.56)	0.053	1.24(0.97-1.59)	0.086	1.23(0.82-1.85)	0.312	1.50(0.91-2.50)	0.114
Q3 (23.13-79.19)	**1.26(1.02-1.56)**	**0.031**	1.19(0.93-1.51)	0.164	1.27(0.88-1.83)	0.197	1.24(0.78-1.97)	0.361
Q4 (≥79.19)	**1.30(1.05-1.61)**	**0.015**	**1.42(1.13-1.80)**	**0.003**	1.25(0.84-1.85)	0.267	**2.06(1.34-3.20)**	**0.001**

HR, Hazard Ratios; 95% CI, 95% Confidence Intervals; O-DMA, O-desmethylangolensin.

Bold values denote statistical significance at the P < 0.05 level.

To determine the effect of phytoestrogens on all-cause and cardiovascular mortality in postmenopausal women, an analysis specifically focusing on women aged >55 years as a distinct subgroup was conducted. Compared with the lowest quartile, an association with reduced risk of all-cause mortality was observed in the third quartile of urinary enterolactone levels (HR = 0.74, 95% CI: 0.57–0.96, *P* = 0.026). Moreover, this association with reduced risk extended to cardiovascular mortality (HR = 0.57, 95% CI: 0.35–0.92, *P* = 0.020). High urinary levels of daidzein (HR = 1.39, 95% CI: 1.08–1.78, *P* = 0.011) and genistein (HR = 1.62, 95% CI: 1.25–2.09, *P* = 0.001) in the highest quartile were associated with increased all-cause mortality. Furthermore, urinary levels of daidzein (HR = 2.12, 95% CI: 1.29–3.46, *P* = 0.003) and genistein (HR = 2.31, 95% CI: 1.44–3.70, *P* = 0.001) in the highest quartile were linked to increased cardiovascular mortality. Notably, urinary O-DMA levels in the highest quartile were a potential risk factor for all-cause mortality (HR = 1.33, 95% CI: 1.03–1.72, *P* = 0.031). The detailed results are presented in **Table 5**.

**Table 5:** The relationships between urinary phytoestrogens levels and all-cause and cardiovascular mortality in the postmenopausal female population.

	All-cause Mortality (HR and 95%CI)		Cardiovascular Mortality (HR and 95%CI)	
	Multivariable Model	*P*	Multivariable Model	*P*
Daidzein (µg/g creatinine)				
Q1 (<16.87)	1 (Reference)		1 (Reference)	
Q2 (16.87-49.19)	0.90(0.69-1.16)	0.410	0.85(0.49-1.48)	0.573
Q3 (49.19-170.08)	1.13(0.88-1.46)	0.338	1.30(0.80-2.11)	0.292
Q4 (≥170.08)	**1.39(1.08-1.78)**	**0.011**	**2.12(1.29-3.46)**	**0.003**
O-DMA (µg/g creatinine)				
Q1 (<0.61)	1 (Reference)		1 (Reference)	
Q2 (0.61-3.00)	1.17(0.90-1.51)	0.246	0.76(0.46-1.27)	0.299
Q3 (3.00-18.93)	0.99(0.76-1.29)	0.954	0.88(0.55-1.43)	0.612
Q4 (≥18.93)	**1.33(1.03-1.72)**	**0.031**	1.31(0.80-2.15)	0.281
Equol (µg/g creatinine)				
Q1 (<3.02)	1 (Reference)		1 (Reference)	
Q2 (3.02-6.61)	1.16(0.89-1.51)	0.272	0.95(0.55-1.64)	0.848
Q3 (6.61-14.15)	0.88(0.67-1.14)	0.336	0.84(0.52-1.33)	0.460
Q4 (≥14.15)	1.07(0.83-1.39)	0.594	1.07(0.66-1.72)	0.778
Enterodiol (µg/g creatinine)				
Q1 (<14.48)	1 (Reference)		1 (Reference)	
Q2 (14.48-38.00)	1.06(0.82-1.38)	0.650	1.27(0.79-2.05)	0.326
Q3 (38.00-91.76)	0.93(0.73-1.19)	0.609	1.07(0.70-1.65)	0.756
Q4 (≥91.76)	1.07(0.83-1.37)	0.594	1.06(0.68-1.65)	0.800
Enterolactone (µg/g creatinine)				
Q1 (<100.24)	1 (Reference)		1 (Reference)	
Q2 (100.24-343.28)	1.06(0.81-1.39)	0.670	0.87(0.54-1.39)	0.568
Q3 (343.28-825.81)	**0.74(0.57-0.96)**	**0.026**	**0.57(0.35-0.92)**	**0.020**
Q4 (≥825.81)	0.92(0.71-1.19)	0.520	0.70(0.45-1.08)	0.110
Genistein (µg/g creatinine)				
Q1 (<8.69)	1 (Reference)		1 (Reference)	
Q2 (8.69-23.13)	1.30(0.99-1.70)	0.055	1.51(0.87-2.63)	0.142
Q3 (23.13-79.19)	1.27(0.98-1.65)	0.070	1.32(0.80-2.19)	0.273
Q4 (≥79.19)	**1.62(1.25-2.09)**	**<0.001**	**2.31(1.44-3.70)**	**0.001**

HR, Hazard Ratios; 95% CI, 95% Confidence Intervals; O-DMA, O-desmethylangolensin.

Bold values denote statistical significance at the P < 0.05 level.

### Dose–response analysis

**Figure 4:**
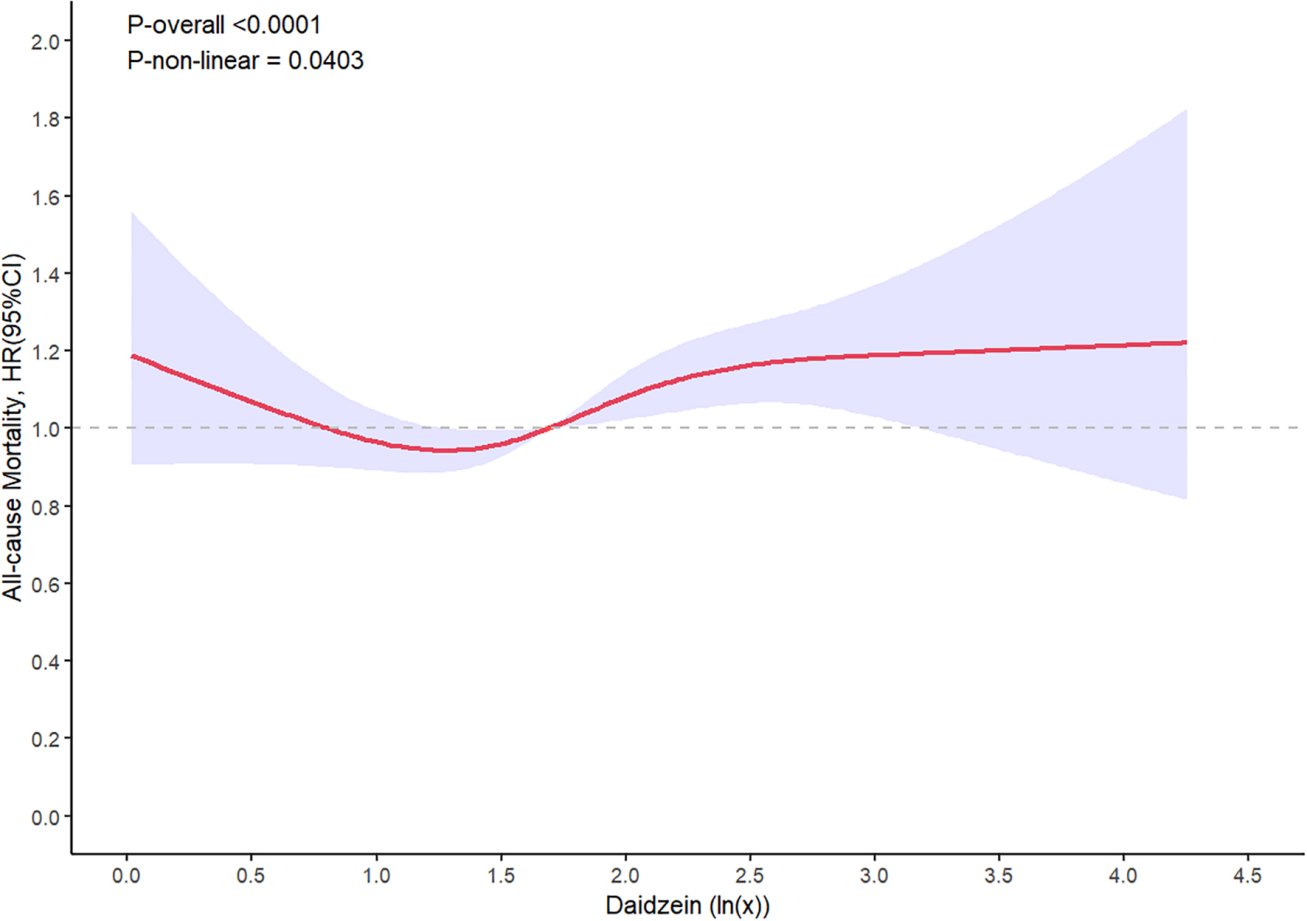
Restricted spline curve shows the relationship between urinary daidzein levels and all-cause mortality. Red line and blue transparent area represent HR and 95% CI, respectively. Data was adjusted for gender, age, race/ethnicity, educational attainment, marital status, family PIR, BMI, waist circumference, heart rates, serum cotinine levels, drinking status, presence of diabetes and hypertension, history of cardiovascular events, serum levels of CRP, TCHO, ALT, GLU, TG, ALB, SCr, and HDL-C.

In the dose–response analysis, the restricted cubic spline demonstrated a meaningful nonlinear relationship between urinary daidzein levels and all-cause mortality (*P*-nonlinearity = 0.04, **Figure 4**). The results indicated a decreased all-cause mortality within the range of corrected urinary daidzein levels of 19.86–46.51 µg/g creatinine and an elevated mortality in the range of 49.23–1482.18 µg/g creatinine. An approximately linear association was observed between all-cause mortality and urinary enterolactone (*P*-nonlinearity = 0.33) and genistein levels (*P*-nonlinearity = 0.29). Furthermore, the HRs for the three urinary phytoestrogen levels related to cardiovascular mortality displayed an approximately linear relationship.

### Sensitivity analysis

A sensitivity analysis was performed by excluding mortality data from the initial 2 years of the follow-up period. Additionally, individuals with preexisting CVD were excluded from the analysis that examined the correlation between urinary phytoestrogens levels and cardiovascular mortality. However, the results remained unchanged (**Supplementary Tables S2, S3**).

## Discussion

In this extensive population-based cohort study, various statistical strategies were utilized for a comprehensive evaluation of the influence of urinary phytoestrogen levels on both all-cause and cardiovascular mortality. The primary findings can be summarized as follows: 1) At least three of the six major phytoestrogens, namely, daidzein, genistein, and enterolactone, were associated with all-cause and cardiovascular mortality. 2) As urinary genistein levels increased, the all-cause and cardiovascular mortality demonstrated a significant upward trend. 3) Although enterolactone appeared to show an association with reduced risks of all-cause and cardiovascular mortality, this association weakened as urinary enterolactone levels increased substantially. 4) Elevated urinary daidzein levels were linked to increased all-cause mortality and exhibited a dose–response relationship. Moreover, urinary daidzein levels were correlated with cardiovascular mortality.

Phytoestrogenic activity in plant extracts was initially noted in 1926 (25). Subsequently, several plants were found to contain phytoestrogens. Detrimental effects of phytoestrogens were first reported in the 1940s. In Australia, certain ewes were observed to suffer from various reproductive disorders, such as delayed conception, infertility, pregnancy-related abortions, developmental abnormalities in the offspring, and ovarian tumors (26). These issues were attributed to their consumption of clover plants rich in phytoestrogens. Subsequently, numerous studies have concentrated on investigating the involvement of phytoestrogens in reproductive toxicity and metabolic disorders. In 1992, a study presented the potential benefits of phytoestrogens and suggested that increased levels of isoflavonoid phytoestrogens might be associated with a reduction in hot flashes and other menopausal symptoms (27). Globally, the quantity of phytoestrogens consumed in diets varies significantly owing to dietary preferences. Particularly in Asian countries where soy-based foods are an integral part of the traditional diet, the daily intake of phytoestrogen-rich isoflavones is 7–25 times higher than that in Western countries (28, 29). Coincidentally, Asians exhibit a lower risk of CVD, menopausal symptoms, hormone-dependent cancers (including breast cancer), DM, and obesity than Westerners. Considering the increased global human consumption of soy and the growing prevalence of phytoestrogen exposure, recognizing the dual impact of phytoestrogens on human health is important.

Phytoestrogens, such as lignans, isoflavones, flavones, coumestans, and stilbenes, are natural compounds that resemble estrogen. Of these, isoflavones and lignans are primary categories. Soybeans is the primary source of isoflavones, and legumes are the next most significant source. Genistein and daidzein, which are members of the isoflavone subgroup, are concentrated in leguminous plants, with soybeans possessing the highest levels. Lignans, the predominant phytoestrogens found in nuts and oilseeds, are likewise distributed across a spectrum of dietary sources, including vegetables, legumes, cereals, and fruits. The absorption and metabolic transformation of isoflavones and lignans heavily depend on the activity of intestinal bacteria (30). Daidzein is metabolized into O-DMA and equol in the gut microbial environment by specific gut bacteria, bacterial combinations, or other contributing factors. Similar to daidzein, multiple bacteria and bacterial consortia are involved in transforming lignans into enterolactone and enterolignan.

Daidzein can be absorbed either directly or after being metabolically transformed into various compounds, such as equol, with high estrogenic activity, or inactive compounds, such as O-DMA (31, 32). Approximately 30%–60% of daidzein is absorbed orally and excreted in the urine, simultaneously engaging in enterohepatic circulation and entering the bile (33). A recent study has shown the presence of an association between daidzein and several diseases, including malignancies, CVD, neurodegenerative disorders, DM, osteoporosis, and dermatological abnormalities (34). This link could be ascribed to the properties of phytoestrogens, which can function as antioxidants, antimutagenics, antiangiogenics, and proapoptotics (35). These functions might be due to the heightened affinity of daidzein for ERβ, thereby enabling the activation of signaling pathways related to ERs. Furthermore, daidzein exerts its effects on numerous signaling pathways unrelated to ERs. Certain studies have reported that soy consumption does not ameliorate disease symptoms or reduce morbidity. In addition, some investigations have observed a genetic signature linked to increased breast cancer cell proliferation in women using soy protein supplements. This finding raises concerns regarding the potential adverse effects for patients with breast cancer (35). While certain studies have indicated an inverse correlation between dietary soy and coronary heart disease, others have failed to discern any such adverse effect (36). These conflicting outcomes could be attributed to dose-dependent effects. The present investigation uncovered a dose–response pattern between the levels of urinary daidzein and all-cause mortality. Levels in the range of 19.86–46.51 µg/g creatinine were correlated with a decrease in all-cause mortality, whereas those in the range from 49.23 to1482.18 µg/g creatinine were linked to an elevated all-cause mortality. Furthermore, the results unequivocally established that increased urinary daidzein levels were a significant risk factor for cardiovascular mortality.

Genistein, the structurally simplest isoflavonoid compound occurring in Leguminosae plants, has attracted immense research interest as a phytoestrogen metabolite. Owing to its molecular weight and lipophilic nature, genistein has limited absorption capacity and is passively transported into intestinal cells. The overall bioavailability of genistein is only approximately 10%, and it is typically excreted via the kidneys within 24 h when ingested orally (37). As a prominent isoflavonoid with a mode of action similar to that of daidzein, genistein preferentially binds to ERβ receptors (38). Consequently, genistein exerts almost the same biological effects as that of daidzein. The current investigation revealed significant connections between urinary genistein levels and all-cause and cardiovascular mortality. Specifically, all-cause mortality was increased when urinary genistein levels fell within the second, third, and fourth quartiles. Unlike the dose–response effect seen with daidzein for all-cause mortality, urinary genistein levels appeared to exhibit a linear and positive correlation with all-cause mortality. This finding indicates that the biological effect of genistein is more potent than that of daidzein, possibly due to its higher affinity for ERs compared with daidzein. Our current study’s results suggest that it is advisable to maintain genistein levels within a low range to mitigate cardiovascular and all-cause mortality.

Enterolactone and enterodiol are the two primary types of lignans. These undergo hydrolysis and deglycosylation within the large intestine, which produces secoisolariciresinol. The unabsorbed secoisolariciresinol is subsequently converted into enterolactone and enterodiol by intestinal bacteria. Once synthesized by intestinal bacteria, these compounds are absorbed and undergo conjugation in the gut epithelium or the liver, typically with sulfate or glucuronic acid, before being excreted via urine and bile. Enterolactone has a longer half-life of approximately 11.6 h compared with enterodiol (approximately 9 h), which makes it the predominant enterolignan in circulation as well as urinary excretion (39). Both enterolactone and enterodiol are antagonistic to ERs. The influence of enterodiol is more pronounced than that of enterolactone. Specifically, enterolactone, but not enterodiol, is implicated in the transcriptional activation of ERβ (40). A study has shown that enterolactone displays stronger enzyme inhibition, including aromatase, 5α-reductase, and 7α-hydroxylase, than enterodiol and enterolactone (41). Furthermore, findings related to the suppression of lipid peroxidation indicate that enterolactone exhibits more potent antioxidant activity (42). While several studies have corroborated the beneficial impact of enterolactone on human health, it is important to note the presence of conflicting findings. Approximately 25 years ago, *The Lancet* published a prospective case–control study that conclusively established the link between elevated serum enterolactone levels in healthy men and a diminished risk of acute coronary events (43). Furthermore, numerous epidemiological studies have documented a decreased risk of many cancers associated with increased dietary lignan exposure, although not all studies support these findings (44). The results from the present study demonstrated that elevated urinary enterolactone levels are linked to a significant reduction in all-cause mortality. The dose–response effect did not achieve significance, but more intricate relationships may exist between urinary enterolactone levels and cardiovascular mortality risk. Specifically, elevated levels within the third quartile were associated with reduced cardiovascular mortality. Nonetheless, this correlation disappeared when the levels reached the fourth quartile.

Given the inconsistent effects of different types of phytoestrogens on human health, studies exploring the relationship between phytoestrogens and various diseases, as well as their mechanisms, have been progressively conducted. However, only a few studies have investigated the relationship between urinary phytoestrogen levels and both all-cause and cardiovascular mortality. Reger and colleagues examined this relationship by analyzing data from NHANES (2009-2014) and NDI mortality records up to December 31, 2006, including 5,179 participants (45). Their findings indicated that elevated urinary daidzein levels were associated with increased cardiovascular mortality, while elevated urinary enterolignan levels were protective against both cardiovascular and all-cause mortality. These results partially align with our findings. However, Reger’s study did not find any significant relationship between urinary genistein levels and either all-cause or cardiovascular mortality. In 2019, Marcelo et al. published a study examining the relationship between urinary genistein levels and both all-cause and cardiovascular mortality, using data from NHANES 2009-2010 (46). They found that elevated urinary genistein levels were associated with an increased risk of both all-cause and cardiovascular mortality. These results are consistent with our findings but differ from those of Reger et al. Theoretically, genistein’s mode of action is similar to that of daidzein, as both preferentially bind to ERβ receptors and exert nearly identical biological effects. Given that elevated urinary daidzein levels are linked to increased all-cause and cardiovascular mortality, it is plausible that genistein may have similar effects. Therefore, we are inclined to support Reger’s findings and those of our current study.

Phytoestrogens have a dual function and either bind to or inhibit ERs, and these effects are modulated by estrogen levels in humans. Consequently, subgroup analyses stratified by sex should be performed. In our present study, urinary daidzein levels were not linked to cardiovascular or all-cause mortality in men. However, the association followed a dose–response pattern in women. Elevated urinary enterolactone levels were linked to decreased all-cause mortality, whereas elevated urinary genistein levels were linked to elevated all-cause mortality. These relationships were observed in both men and women, with a notably stronger correlation in women. In men, cardiovascular mortality was not associated with urinary genistein or enterolactone levels, whereas these associations remained significant in women. These disparities in the associations between phytoestrogens and all-cause and cardiovascular mortality in two sexes could be attributed to variations in ERs between men and women. Estrogen levels and ER sensitivity decline in postmenopausal women. Consequently, a stratified analysis was conducted to explore the associations between urinary phytoestrogen levels and all-cause and cardiovascular mortality in postmenopausal women. The findings closely resembled those of the overall population of women, except for a negligible association between urinary O-DMA levels and all-cause mortality.

This study has several limitations. First, the study is that while we observed associations between urinary phytoestrogen levels and mortality risks, we cannot directly recommend interventions based on these findings. Our observational study design precludes establishing causality. The relationships between phytoestrogens and health outcomes are complex, involving non-linear associations, individual variations in metabolism, and potential unintended effects. Before developing operable interventions, further research is needed, including replication studies, mechanistic investigations, and controlled trials. Additionally, any future interventions may need to be personalized due to individual differences in phytoestrogen metabolism and response. Second, phytoestrogens were evaluated using a single measurement, which failed to capture the average level over a period of time. Measuring phytoestrogen levels shortly after consuming phytoestrogen-rich foods can lead to elevated urinary levels and misclassification. Third, dietary information was not included in the study. Although dietary data can be obtained from NHANES, wide variations in the phytoestrogen content of foods posed challenges in its accurate qualitative and quantitative determination. Fourth, these findings were based on the U.S. adult population, which may limit their generalizability to other demographic groups. Lastly, although a significant decrease in cardiovascular mortality was observed when urinary enterolactone levels were in the third quartile, this association diminished as the levels continued to rise. Despite conducting a dose–response analysis that did not yield statistical significance, residual and unmeasured confounding factors cannot be completely ruled out.

The results suggest that elevated urinary enterolactone levels were associated with reduced risk of both all-cause and cardiovascular mortality. On the contrary, increased urinary genistein levels were linked to increased all-cause and cardiovascular mortality. These levels demonstrated a dose–response relationship with all-cause mortality and were associated with cardiovascular mortality. The relationship between urinary phytoestrogen levels and all-cause and cardiovascular mortality exhibited some variations between men and women, but these associations remained relatively consistent in both premenopausal and postmenopausal women. The precise physiological functions of most phytoestrogens remain incompletely understood. Consequently, caution should be exercised when interpreting the effects observed in epidemiological studies.

## Data Availability

Publicly available datasets were analyzed in this study. This data can be found here: National Health and Nutrition Examination Survey.
