# Supplementary material for: Associations of urinary phytoestrogens with all-cause and cardiovascular mortality in adults: a population-based cohort study
Source: Front Endocrinol (Lausanne). 2024 Sep 10;15:1400182. doi: 10.3389/fendo.2024.1400182 (PMC11419972; doi:10.3389/fendo.2024.1400182)
Supplement: Supplementary file 2 [file Table2.docx]

| **Table S2.** Sensitivity analysis between urinary phytoestrogens concentrations and risk of all-cause and Cardiovascular Mortality by excluding mortality data from the initial two years of the follow-up period. | | | | |
| --- | --- | --- | --- | --- |
|  | **All-cause Mortality (HR and 95%CI)** | | **Cardiovascular Mortality (HR and 95%CI)** | |
|  | **Multivariable Model** | ***P*** | **Multivariable Model** | ***P*** |
| [Daidzein](https://wwwn.cdc.gov/Nchs/Nhanes/1999-2000/PHPYPA.htm)  [(µg/g cratinine)](https://wwwn.cdc.gov/Nchs/Nhanes/1999-2000/PHPYPA.htm) |  |  |  |  |
| Q1 (<16.87) | 1[Reference] |  | 1[Reference] |  |
| Q2 (16.87-49.19) | 0.92(0.78-1.09) | 0.338 | 0.83(0.60-1.15) | 0.262 |
| Q3 (49.19-170.08) | 0.96(0.82-1.13) | 0.643 | 0.82(0.61-1.11) | 0.200 |
| Q4 (≥170.08) | **1.18(1.00-1.38)** | **0.045** | **1.47(1.09-1.97)** | **0.011** |
| [O-DMA](https://wwwn.cdc.gov/Nchs/Nhanes/1999-2000/PHPYPA.htm)  [(µg/g cratinine)](https://wwwn.cdc.gov/Nchs/Nhanes/1999-2000/PHPYPA.htm) |  |  |  |  |
| Q1 (<0.61) | 1[Reference] |  | 1[Reference] |  |
| Q2 (0.61-3.00) | 1.01(0.85-1.19) | 0.925 | **0.70(0.51-0.97)** | **0.033** |
| Q3 (3.00-18.93) | 1.01(0.85-1.19) | 0.37 | 0.92(0.69-1.22) | 0.544 |
| Q4 (≥18.93) | 1.10(0.94-1.30) | 0.236 | 1.12(0.83-1.50) | 0.450 |
| [Equol](https://wwwn.cdc.gov/Nchs/Nhanes/1999-2000/PHPYPA.htm)  [(µg/g cratinine)](https://wwwn.cdc.gov/Nchs/Nhanes/1999-2000/PHPYPA.htm) |  |  |  |  |
| Q1 (<3.02) | 1[Reference] |  | 1[Reference] |  |
| Q2 (3.02-6.61) | 0.97(0.82-1.15) | 0.711 | 0.96(0.69-1.35) | 0.829 |
| Q3 (6.61-14.15) | 0.90(0.76-1.06) | 0.213 | 0.86(0.63-1.17) | 0.338 |
| Q4 (≥14.15) | 0.96(0.81-1.14) | 0.659 | 0.99(0.72-1.34) | 0.925 |
| [Enterodiol](https://wwwn.cdc.gov/Nchs/Nhanes/1999-2000/PHPYPA.htm)  [(µg/g cratinine)](https://wwwn.cdc.gov/Nchs/Nhanes/1999-2000/PHPYPA.htm) |  |  |  |  |
| Q1 (<14.48) | 1[Reference] |  | 1[Reference] |  |
| Q2 (14.48-38.00) | 0.96(0.82-1.13) | 0.640 | 1.01(0.75-1.35) | 0.970 |
| Q3 (38.00-91.76) | 0.87(0.74-1.02) | 0.091 | 0.82(0.61-1.09) | 0.175 |
| Q4 (≥91.76) | 1.01(0.85-1.19) | 0.939 | 0.91(0.67-1.24) | 0.542 |
| [Enterolactone](https://wwwn.cdc.gov/Nchs/Nhanes/1999-2000/PHPYPA.htm)  [(µg/g cratinine)](https://wwwn.cdc.gov/Nchs/Nhanes/1999-2000/PHPYPA.htm) |  |  |  |  |
| Q1 (<100.24) | 1[Reference] |  | 1[Reference] |  |
| Q2 (100.24-343.28) | 0.92(0.77-1.09) | 0.345 | 0.95(0.69-1.29) | 0.729 |
| Q3 (343.28-825.81) | **0.78(0.66-0.92)** | **0.004** | 0.77(0.56-1.04) | 0.092 |
| Q4 (≥825.81) | **0.85(0.72-1.00)** | **0.046** | 0.85(0.63-1.15) | 0.286 |
| [Genistein](https://wwwn.cdc.gov/Nchs/Nhanes/1999-2000/PHPYPA.htm)  [(µg/g cratinine)](https://wwwn.cdc.gov/Nchs/Nhanes/1999-2000/PHPYPA.htm) |  |  |  |  |
| Q1 (<8.69) | 1[Reference] |  | 1[Reference] |  |
| Q2 (8.69-23.13) | **1.24(1.04-1.47)** | **0.016** | 1.29(0.93-1.78) | 0.134 |
| Q3 (23.13-79.19) | **1.24(1.05-1.47)** | **0.011** | 1.24(0.92-1.68) | 0.164 |
| Q4 (≥79.19) | **1.33(1.13-1.57)** | **0.001** | **1.55(1.15-2.08)** | **0.004** |

**HR:** Hazard Ratios; **95% CI:** 95% Confidence Intervals; **O-DMA:** O-desmethylangolensin.
