# Supplementary material for: Associations of urinary phytoestrogens with all-cause and cardiovascular mortality in adults: a population-based cohort study
Source: Front Endocrinol (Lausanne). 2024 Sep 10;15:1400182. doi: 10.3389/fendo.2024.1400182 (PMC11419972; doi:10.3389/fendo.2024.1400182)
Supplement: Supplementary file 3 [file Table3.docx]

| **Table S3.** Sensitivity analysis between urinary phytoestrogens concentrations and risk of Cardiovascular Mortality. | | |
| --- | --- | --- |
|  | **Cardiovascular Mortality (HR and 95%CI)** | |
|  | **Multivariable Model** | ***P*** |
| [Daidzein (µg/g cratinine)](https://wwwn.cdc.gov/Nchs/Nhanes/1999-2000/PHPYPA.htm) |  |  |
| Q1 (<16.87) | 1[Reference] |  |
| Q2 (16.87-49.19) | 0.84(0.55-1.27) | 0.402 |
| Q3 (49.19-170.08) | 0.97(0.67-1.39) | 0.857 |
| Q4 (≥170.08) | **1.71(1.19-2.47)** | **0.004** |
| [O-DMA (µg/g cratinine)](https://wwwn.cdc.gov/Nchs/Nhanes/1999-2000/PHPYPA.htm) |  |  |
| Q1 (<0.61) | 1[Reference] |  |
| Q2 (0.61-3.00) | 0.71(0.48-1.05) | 0.090 |
| Q3 (3.00-18.93) | 0.89(0.63-1.26) | 0.511 |
| Q4 (≥18.93) | 1.17(0.83-1.65) | 0.375 |
| [Equol (µg/g cratinine)](https://wwwn.cdc.gov/Nchs/Nhanes/1999-2000/PHPYPA.htm) |  |  |
| Q1 (<3.02) | 1[Reference] |  |
| Q2 (3.02-6.61) | 1.07(0.73-1.58) | 0.728 |
| Q3 (6.61-14.15) | 0.78(0.55-1.11) | 0.167 |
| Q4 (≥14.15) | 1.08(0.75-1.56) | 0.674 |
| [Enterodiol (µg/g cratinine)](https://wwwn.cdc.gov/Nchs/Nhanes/1999-2000/PHPYPA.htm) |  |  |
| Q1 (<14.48) | 1[Reference] |  |
| Q2 (14.48-38.00) | 1.06(0.74-1.53) | 0.735 |
| Q3 (38.00-91.76) | 0.94(0.67-1.33) | 0.739 |
| Q4 (≥91.76) | 0.98(0.67-1.42) | 0.914 |
| [Enterolactone (µg/g cratinine)](https://wwwn.cdc.gov/Nchs/Nhanes/1999-2000/PHPYPA.htm) |  |  |
| Q1 (<100.24) | 1[Reference] |  |
| Q2 (100.24-343.28) | 0.80(0.56-1.15) | 0.225 |
| Q3 (343.28-825.81) | **0.55(0.38-0.80)** | **0.002** |
| Q4 (≥825.81) | 0.80(0.56-1.16) | 0.239 |
| [Genistein (µg/g cratinine)](https://wwwn.cdc.gov/Nchs/Nhanes/1999-2000/PHPYPA.htm) |  |  |
| Q1 (<8.69) | 1[Reference] |  |
| Q2 (8.69-23.13) | **1.56(1.03-2.36)** | **0.036** |
| Q3 (23.13-79.19) | **1.65(1.13-2.41)** | **0.009** |
| Q4 (≥79.19) | **1.95(1.35-2.81)** | **<0.001** |

**HR:** Hazard Ratios; **95% CI:** 95% Confidence Intervals; **O-DMA:** O-desmethylangolensin.
